# Lignosulfonate-mediated cellulase adsorption: enhanced enzymatic saccharification of lignocellulose through weakening nonproductive binding to lignin

**DOI:** 10.1186/1754-6834-6-156

**Published:** 2013-11-05

**Authors:** Zhaojiang Wang, JY Zhu, Yingjuan Fu, Menghua Qin, Zhiyong Shao, Jungang Jiang, Fang Yang

**Affiliations:** 1 Key Laboratory of Paper Science and Technology, Ministry of Education, Qilu University of Technology, Jinan, China; 2 US Forest Service, Forest Products Laboratory, Madison, WI, USA; 3 Laboratory of Organic Chemistry, Taishan University, Tai’an 271021, China

**Keywords:** Enzymatic saccharification/hydrolysis, Lignin/lignosulfonate, Nonproductive/nonspecific binding/adsorption, Polyelectrolyte-protein complexes, Pretreatment

## Abstract

**Background:**

Thermochemical pretreatment of lignocellulose is crucial to bioconversion in the fields of biorefinery and biofuels. However, the enzyme inhibitors in pretreatment hydrolysate make solid substrate washing and hydrolysate detoxification indispensable prior to enzymatic hydrolysis. Sulfite pretreatment to overcome recalcitrance of lignocelluloses (SPORL) is a relatively new process, but has demonstrated robust performance for sugar and biofuel production from woody biomass in terms of yield and energy efficiency. This study demonstrated the advantage of SPORL pretreatment whereby the presentation of lignosulfonate (LS) renders the hydrolysate non-inhibitory to cellulase (Cel) due to the formation of lignosulfonate-cellulase complexes (LCCs) which can mediate the Cel adsorption between lignin and cellulose, contrary to the conventional belief that pretreatment hydrolysate inhibits the enzymatic hydrolysis unless detoxified.

**Results:**

Particular emphasis was made on the formation mechanisms and stability phase of LCCs, the electrostatic interaction between LCCs and lignin, and the redistributed Cel adsorption between lignin and cellulose. The study found that LS, the byproduct of SPORL pretreatment, behaves as a polyelectrolyte to form LCCs with Cel by associating to the oppositely charged groups of protein. Compared to Cel, the zeta potential of LCCs is more negative and adjustable by altering the molar ratio of LS to Cel, and thereby LCCs have the ability to mitigate the nonproductive binding of Cel to lignin because of the enlarged electrostatic repulsion. Experimental results showed that the benefit from the reduced nonproductive binding outweighed the detrimental effects from the inhibitors in pretreatment hydrolysate. Specifically, the glucan conversions of solid substrate from poplar and lodgepole pine were greatly elevated by 25.9% and 31.8%, respectively, with the complete addition of the corresponding hydrolysate. This contradicts the well-acknowledged concept in the fields of biofuels and biorefinery that the pretreatment hydrolysate is inhibitory to enzymes.

**Conclusions:**

The results reported in this study also suggest significant advantages of SPORL pretreatment in terms of water consumption and process integration, that is, it should abolish the steps of solid substrate washing and pretreatment hydrolysate detoxification for direct simultaneous saccharification and combined fermentation (SSCombF) of enzymatic and pretreatment hydrolysate, thereby facilitating bioprocess consolidation. Furthermore, this study not only has practical significance to biorefinery and bioenergy, but it also provides scientific importance to the molecular design of composite enzyme-polyelectrolyte systems, such as immobilized enzymes and enzyme activators, as well as to the design of enzyme separation processes using water-soluble polyelectrolytes.

## Background

Lignocellulose represents a key sustainable source of biomass for transformation into biofuels and bio-based products. However, the lignin-polysaccharide cross-linking structure of lignocellulose makes it recalcitrant to biotransformation, both microbial and enzymatic. For this reason, thermochemical strategies are widely implemented to make lignocellulose accessible to cellulase (Cel) by liberating polysaccharides from the lignin seal [1]. Considering the protection of sugars from undesired degradation, it is impossible to attain the complete removal of lignin for the existing thermochemical pretreatments, especially for feedstocks with high lignin content, such as forest biomass [2]. The residual lignin in solid substrate tends to adsorb the enzyme nonproductively and irreversibly during enzymatic hydrolysis, not only violating the selectivity of enzymatic catalysis, but also leading to enzyme deactivation [3,4]. Furthermore, the thermochemical pretreatment invariably involves the degradation of lignocellulose. Some byproducts of pretreatment may act as inhibitors to enzymes and microorganisms, such as furfural and hydroxymethylfurfural (HMF), which are regarded as the most toxic inhibitors present in pretreatment hydrolysate [5]. In this context, the washing of solid substrate and detoxification of pretreatment hydrolysate have to be indispensably performed prior to enzymatic hydrolysis or fermentation, which not only result in tremendous consumption of water and energy, but also complicate the bioconversion process. On the whole, the nonproductive binding and inhibitory effects are the main obstacles to the sustainable development of biofuels in the upstream processes of bioconversion.

Various approaches have been applied to reduce the nonproductive Cel binding, including genetic [6,7] and chemical [8] modification of lignin, specific lignin removal [9,10], and lignin shielding by BSA [11] or surfactant [12,13]. These approaches are somewhat effective in mitigating the enzyme-lignin interaction, but further complicate the process and make the bioconversion unprofitable due to the additional expense. To liberate Cel molecules from the effects of lignin, we proposed sulfite pretreatment to overcome recalcitrance of lignocellulose (SPORL), as reported previously [14]. Recently, efforts contributed to the study of lignin modification and the resultant reduction of the nonproductive binding. Firstly, sulfonation modification occurs to lignin during SPORL pretreatment, which makes lignin negatively charged. Because the ionization of the sulfonic acid group on lignin is pH-dependent, elevating pH was observed to be effective to reduce the nonproductive binding. This mechanism was recently reported by Lan *et al*. [15], which is similar to the mechanism proposed by Del Rio that the sulfonation of lignin was effective for cellulolytic hydrolysis improvement due to the increase of free enzymes [16]. Secondly, lignosulfonate (LS), a byproduct of SPROL in pretreatment hydrolysate, has a unique irregular structure with a non-charged core consisting of cross-linked aromatic chains with all sulfonic acid groups relocated to or near the molecule surface to facilitate interactions with the aqueous surroundings. Although the overall structure of LS is not known, it possesses the same ionizable groups as polyacrylamidomethylpropyl sulfonate (PAMPS), an anionic polyelectrolyte that can bind BSA and form a complex, as reported by Mattison [17]. Therefore, we hypothesize that the LS is likely to associate and form LS-Cel complexes (LCCs) with Cel by acting as a polyelectrolyte. If this is the case, the resultant LCCs will be more negatively charged, and thereby the electrostatic repulsive force between lignin and enzymes (LCCs) will become stronger. This will result in the redistribution of Cel adsorption on lignin and cellulose, and definitely mitigate the nonproductive binding and promote the enzymatic saccharification of lignocellulosic substrate. Furthermore, such interaction is supposed to be adjustable since the surface charge of LCCs is dependent on the proportion of LS at a given pH value. In this case, the pretreatment hydrolysate that was considered to be inhibitory to enzymes and yeast [5,18] may not have to be detoxified prior to enzymatic saccharification or fermentation, provided the constructive effects from LCCs outweigh the detrimental effects from inhibitors in the pretreatment hydrolysate. In fact, our latest study showed that up to 90% of cellulose saccharification was achieved for SPORL-pretreated lodgepole pine at Cel loading of 13 FPU/g glucan with the application of its corresponding pretreatment hydrolysate coupled with increasing enzymatic hydrolysis pH to above 5.5, compared with only 51% for the control run without hydrolysate application at pH 5.0 [19]. This provides great advantage for SPORL in terms of water consumption and process consolidation. However, we ascribed the enhanced enzymatic saccharification to the role of LS as surfactant in the last study [19], rather than the role of water-soluble anionic polyelectrolyte that is now supposed to form complexes with Cel.

The interactions between polyelectrolytes and proteins have been extensively studied in a variety of contexts, such as DNA replication and gene regulation [20], protein purification [21,22] and separation [23], enzyme immobilization [24,25] and activity control [26,27], and polymer-mediated drug delivery [28,29]. Since LS can interact with proteins, it has been exploited as a precipitator for protein recovery [30,31], a bypass protein for ruminants to improve nutrition uptake [32,33], and an organic fertilizer for maize metabolism by enhancing enzyme activity [34]. However, the interaction between LS and Cel has not received enough attention in the aspect of biofuel production. The present study was carried out to clarify the formation of LCCs, and elucidate its role of mediating the Cel adsorption between lignin and cellulose. In addition, the effects of LCCs on enzymatic saccharification of lignocellulose were evaluated.

## Results and discussion

### Interactions between Cel and LS

**Figure 1:**
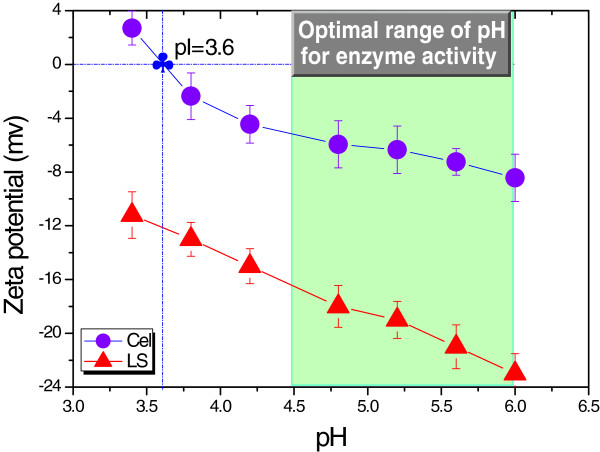
**pH-dependent net surface charge of Cel and LS.** Zeta potential was measured at 25°C using laser Doppler microelectrophoresis. The 0.1 M H_2_SO_4_ and 0.1 M NaOH were used for pH adjustment prior to measurements. Cel, cellulase; LS, lignosulfonate.

**Figure 2:**
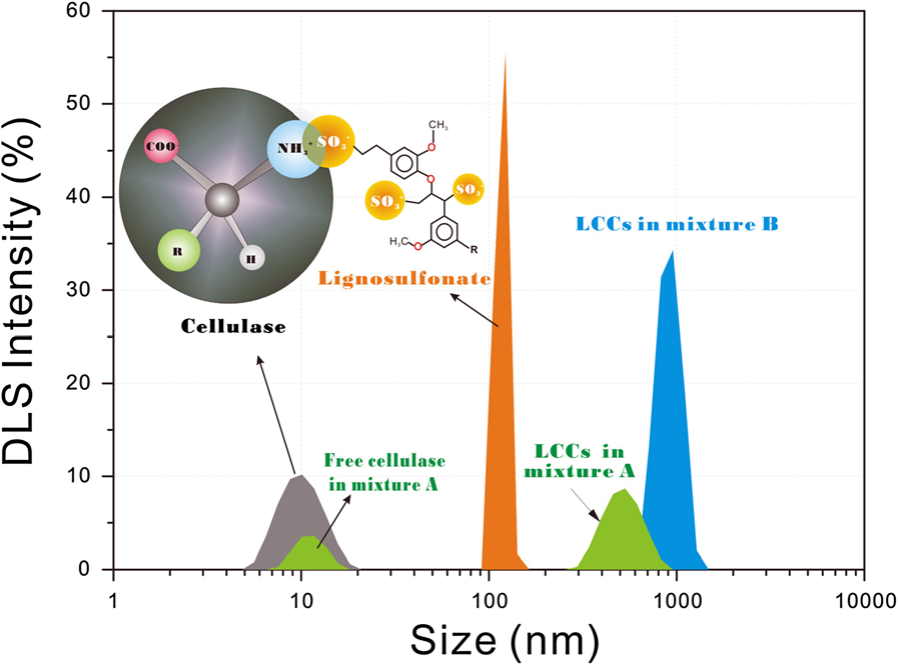
**Intensity size distributions for Cel (1.5 mg/mL, MW = 50 kDa), LS (3.4 mg/mL, MW = 4,200 Da), and LCCs in mixtures.** The molar ratio of Cel to LS is 10^4^/3 in mixture A (800 μL Cel and 10 μL LS), and 10^4^/20 in mixture B (800 μL Cel and 60 μL LS). DLS analysis was conducted in 50 mM sodium acetate buffer at pH 4.8 and 25°C after the required time of 60 minutes for equilibrium. The figure insert is the schematic presentation of the formation of LCCs. Cel, cellulase; DLS, dynamic light scattering; LCC, lignosulfonate-cellulase complex; LS, lignosulfonate; MW, molecular weight.

Cel, a protein-based amphoteric molecule in nature, contains both acidic and basic functional groups. The net charge on Cel is affected by the pH of its surrounding environment, and can become more positively or negatively charged due to the loss or gain of protons (Figure 1). The isoelectric point (pI) of Cel was attained at pH 3.6. The separated LS from SPROL hydrolysate, which possesses sulfonic acid groups, has an electronegative potential that is far less than that of Cel at the same pH. Similarly, the net charge of LS is also dependent on the pH of the solution as shown in Figure 1. In the optimal range of pH for Cel activity from 4.5 to 6.0, both Cel and LS are negatively charged, and thereby the nonflexible Cel molecules can only associate with the flexible LS through the stoichiometric formation of ion pairs, that is, the integration of the amino group in Cel and sulfonic acid group in LS, as the schematic representation demonstrates in Figure 2. To verify the complexation of LS and Cel, LS solution was mixed with Cel solution at pH 4.8. The mixture was then subjected to dynamic light scattering (DLS) analysis after equilibrium. Figure 2 shows the size distributions of Cel, LS, and LCCs in mixtures with different molar ratios of Cel to LS. The unimodal distribution with an apparent diameter of 10 nm corresponds to Cel, and the peak at 126 nm represents LS. For mixture A with excess Cel (Cel/LS = 10^4^/3), the negative charge on LS was not sufficient to capture the Cel molecules as indicated by the peaks at 10 nm and 385 nm, which correspond to the free Cel and LCCs, respectively. The increased fraction of LS in mixture B (Cel/LS = 10^4^/20) resulted in total complexation as indicted by the only peak of LCCs with larger size at 937 nm (Figure 2). From another perspective, the complexation can be considered as enzyme immobilization just as Haupt *et al*. reported that glucoamylase and β-glucosidase were immobilized on a novel type of colloidal particle consisting of a poly(styrene) core and long chains of poly(styrene sulfonic acid) brush [35].

**Figure 3:**
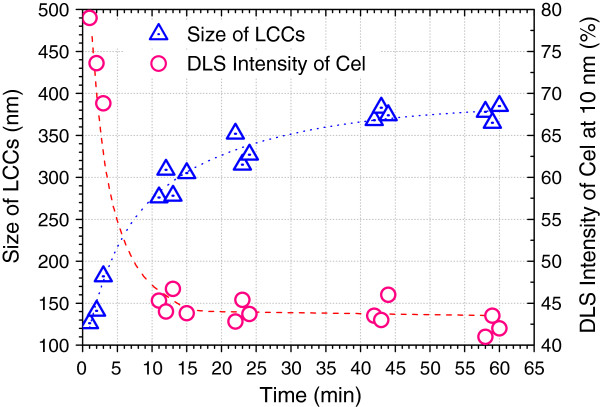
**Process of the formation of LCCs in mixture A (10 μL LS, 3.4 mg/mL, and 800 μL Cel, 1.5 mg/mL).** DSL analysis was conducted at pH 4.8 and 25°C. Cel, cellulase; DSL, dynamic light scattering; LCC, lignosulfonate-cellulase complex; LS, lignosulfonate.

Since LS behaves as a branched polyelectrolyte with flexible negatively charged chains, the entrapment of Cel by the chains of LS is considered to be efficient and fast. Figure 3 provides information about the process of the formation of LCCs in mixture A. The entrapment was accomplished within 15 minutes as indicated by DLS intensity of Cel at 10 nm, but the LCCs kept dimensionally growing after the accomplishment of entrapment until reaching equilibrium at 60 minutes. This can be interpreted by the time-consuming process of encapsulation of Cel molecules by the clench of LS chains.

**Figure 4:**
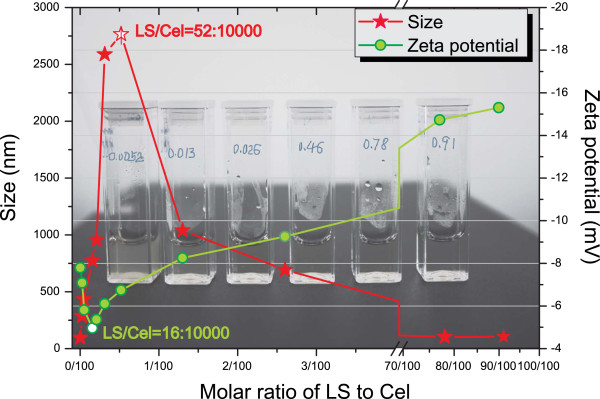
**LS/Cel dependence of size and zeta potential for the LCCs in 50 mM buffer at pH 4.8 and 25°C.** Various molar ratios of LS to Cel were realized by adding LS to 100 μL of Cel solution (1.5 mg/mL). The background image shows the mixtures with various molar ratios of LS to Cel at equilibrium. Cel, cellulase; LCC, lignosulfonate-cellulase complex; LS, lignosulfonate.

Since the LCCs are dimensionally larger than Cel molecules, stronger electrostatic repulsion among particles was required to stabilize the solution of LCCs. Otherwise the LCCs would evolve into larger clusters, and eventually coacervate and precipitate. This would be fatal for the enzymatic catalysis of solid lignocellulosic substrate. Therefore, particular emphasis was made on the stability phase of LCCs at various molar ratios of LS to Cel. Figure 4 shows the size and zeta potential of LCCs in a broad range of LS/Cel, from approximately 1/10^5^ to 1/1. As expected, the addition of LS to Cel solution led to the coacervation and precipitation at 52/10^4^ of LS/Cel, where the size attained the maximum value of 2,765 nm. Moreover, the reverse process, referred to as redissolution [36], appeared upon further addition of LS as a result of the decrescent particle size and more negative surface potential. Given the size of the redissolved LCCs was about 105 nm, approximately the same as LS, the redissolved LCCs can be considered to be micelles with the flexible chains of LS carrying Cel molecules. Away from the main subject of the present study, the LS can also be used for enzyme separation because the coacervate of LCCs appeared at a very low molar ratio of LS to Cel, as shown in Figure 4. Furthermore, the separated Cel is supposed to be easy to use, since the further addition of LS makes it soluble again. The results reported here showed some similarity to the study of Lu *et al*. [37], who reported that the anionic polyelectrolyte in low concentration can promote nucleation when they are utilized as the precipitants for protein separation.

### LCCs-induced reduction of nonproductive Cel binding to lignin

**Figure 5:**
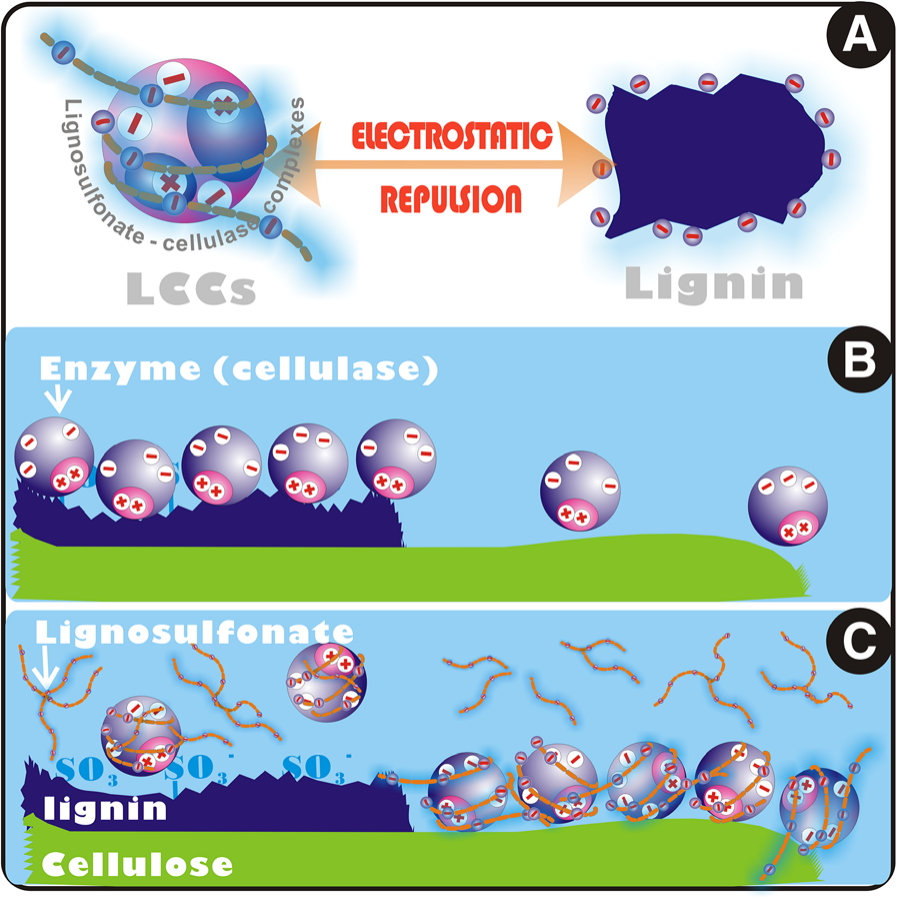
**Schematic representation of LCCs-mediated Cel adsorption between lignin and cellulose. (A)** Greater electrostatic repulsion between Cel and lignin is derived from the formation of LCCs that possess greater negative potential. **(B)** Binding of Cel to lignin in the case of enzymatic hydrolysis of lignocellulosic substrate without LS application. **(C)** Reduced Cel binding to lignin due to the LS application, which promotes the selectivity of enzymatic catalysis. Cel, cellulase; LCC, lignosulfonate-cellulase complex; LS, lignosulfonate.

**Figure 6:**
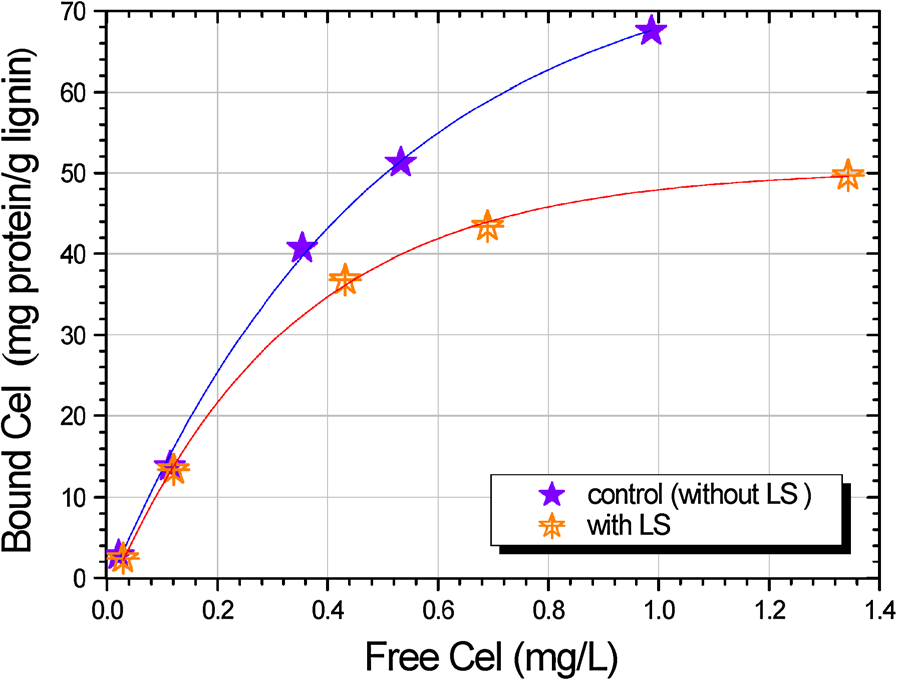
**Isotherms of Cel binding to lignin with and without LS application at 25°C and pH 5.2.** The lignin was the hydrolysis lignin residuals from solid substrate SP-Po-A_1_B_4_ (see Table 3 for details about SP-Po-A_1_B_4_). Cel, cellulase; LS, lignosulfonate; SPORL, sulfite pretreatment to overcome recalcitrance of lignocellulose.

The elevated surface charge of LCCs is not only constructive to the stability of the solution of LCCs, but results in stronger electrostatic repulsion force between Cel and lignin, thus weakening the nonproductive binding of Cel to lignin. Consequently, the Cel binding is redistributed between lignin and cellulose during enzymatic hydrolysis of lignocellulose, which leads to the elevated catalytic selectivity. This mechanism is demonstrated in Figure 5. To verify the LCCs’ function of nonproductive binding reduction, the pure lignin (referred to as hydrolysis lignin residues) was made by repeated enzymatic hydrolysis of lignocellulosic substrate from SPORL-pretreated poplar. The Cel adsorption on the hydrolysis lignin residues was measured in the range of 0 to 1.4 mg/mL protein (Figure 6). The isotherms of Cel binding clearly showed the reduction of bound Cel as a result of LS application. To further quantify the nonproductive Cel binding, linear fitting was made to all experimental data collected to examine the maximum binding capacity Q_s_ by using the Langmuir model equation (1). The value of Q_s_ was reduced from 182.92 to 88.5 mg protein/g lignin as a result of LS application (Table 1), which suggested the significant role of LS in weakening the nonproductive binding. In fact, many approaches have been carried out with the purpose of elimination of nonproductive binding, including transgenic technology to reduce the lignin content [6], addition of additives (BSA, polyethylene glycol, and Tween 20) [38], and chemical modification of lignin [8]. The study by Eriksson *et al*. revealed that the non-ionic surfactants were most effective to nonproductive binding reduction in which the approximate reduction of enzyme adsorption was from 90% adsorbed enzyme to 80% with surfactant addition [12]. Similar results were also reported by Borjesson *et al*. by using poly(ethylene glycol) as additives [39]. The LS reported in the present study can be considered as additives that have a positive effect on nonproductive binding reduction. Since LS is the byproduct of pretreatment, this will undoubtedly promote the economical feasibility of biofuel production.

**Table 1:** Results from linear regressions of Cel adsorption isotherms by hydrolysis lignin residues using the Langmuir model

	***k *** **(mL/mg protein)**	**Q**_**s **_**(mg protein/g lignin)**	**r**^**2**^
Without LS	0.72	182.92	0.99
With LS	1.44	88.50	0.99

Cel, cellulase; LS, lignosulfonate.

### Enzymatic hydrolysis of SPORL-pretreated substrates with the application of SPORL pretreatment hydrolysate

**Figure 7:**
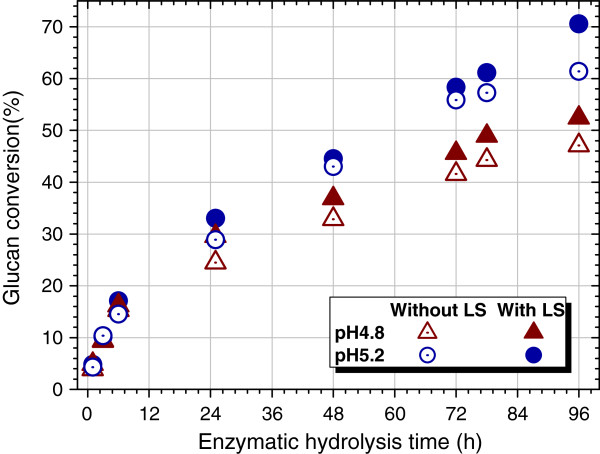
**Effect of LS (8 mL, 4.5 mg/mL, from its corresponding pretreatment) on enzymatic saccharification of substrate SP-Po-A**_**1**_**B**_**4 **_**at pH 4.8 and pH 5.2.Cel 7.5 FPU/g glucan and β-glucosidase 11.25 CBU/g glucan were used for enzymatic hydrolysis (2.0 g solid substrate).** Cel, cellulase; LS, lignosulfonate.

After verification of the reduced nonproductive binding, the concern is extended to whether the biochemical activity of Cel is maintained in the resulting complexes; the answer to which is central to the molecular design of composite enzyme-polyelectrolyte systems, such as enzyme stabilizers and activators, as well as to the design of enzyme separation processes using water-soluble polyelectrolytes. To answer this question, LS was mixed with solid substrate SP-Po-A_1_B_4,_ and then subjected to enzymatic saccharification. As expected, the terminal glucan conversion at 96 hours was elevated by 5% and 9% at pH 4.8 and pH 5.2, respectively (Figure 7). It can be deduced that the interactions between LS and Cel and the resultant complexes do not inhibit the enzyme activity. The enhancement of glucan conversion is considered to be the profit of the LS-induced reduction of nonproductive binding. For dilute acid pretreatment without LS production, the glucan conversion at the same Cel loading was much lower, in the range from approximately 21% to 49%, as shown in our previous work [40]. The greater gain of glucan conversion obtained at pH 5.2 can be ascribed to the pH-induced change of the surface potential of lignin, reported by Lan *et al*. [15]. The above results indicated that LCCs maintained full activity. Similar results were reported in a study on immobilization of β-glucosidase to spherical polyelectrolyte brushes (polystyrene sulfonic acid) [35]. Such protection of enzyme activity was ascribed to the reduction of thermal deactivation [41] and surface deactivation [42] when using surfactants as an activity protector. LS is a type of surfactant that is derived from sulfonation of lignin, and it is likely that LS protects the enzymes from deactivation during hydrolysis.

**Figure 8:**
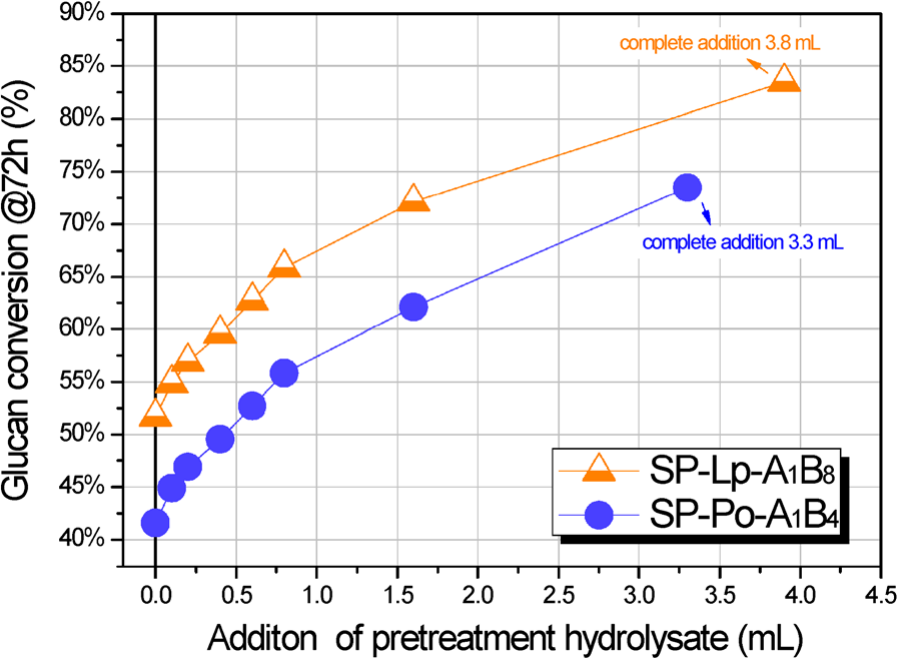
**Effect of the pretreatment hydrolysate on glucan conversion of solid substrate SP-Lp-A**_**1**_**B**_**8 **_**and SP-Po-A**_**1**_**B**_**4**_**.** (See Table 3 for details about SP-Lp-A_1_B_8_ and SP-Po-A_1_B_4_). The enzymatic hydrolysis was conducted at pH 4.8 with Cel dosage of 15 FPU/g glucan and β-glucosidase dosage of 22.5 CBU/g glucan for SP-Lp-A_1_B_8_ (0.8 g solid substrate), and 7.5 FPU/g glucan and β-glucosidase 11.25 CBU/g glucan for SP-Po-A_1_B_4_ (0.8 g solid substrate). Cel, cellulase; SPORL, sulfite pretreatment to overcome recalcitrance of lignocellulose.

Besides LS, the SPORL hydrolysate also contains degradation products (Table 2). Some of the products are enzyme inhibitors, particularly furfural and HMF, which are regarded as the most toxic inhibitors present in pretreatment hydrolysate [43,44]. To examine the combined effects of LS and inhibitors on glucan conversion of solid substrates, the SPORL hydrolysate was directly applied to enzymatic hydrolysis of solid substrates SP-Lp-A_1_B_8_ (SPORL-pretreated lodgepole pine) and SP-Po-A_1_B_4_ (SPORL-pretreated poplar). As shown in Figure 8, the striking increases of glucan conversion at 72 hours were observed with the addition of pretreatment hydrolysate, indicating that the benefits from LS outweigh the loss from inhibitors. Furthermore, the enhancement was almost linearly correlated to the addition of pretreatment hydrolysate. Considering the wood to liquor ratio of 1:3 (w/v) and the solid substrate yields of pretreatment (73.5% for poplar and 62.2% for lodgepole pine) in Table 2, the amounts of pretreatment hydrolysate (3.3 mL for SPORL-Po-A_1_B_4_ and 3.8 mL for SP-Lp-A_1_B_8_) in Figure 8 correspond to the amount of solid substrate used for enzymatic hydrolysis, namely 0.8 g. This means that the complete addition of pretreatment hydrolysate leads to the remarkable enhancement in glucan conversion; contrary to the conventional understanding that pretreatment hydrolysate inhibits the enzymatic hydrolysis unless detoxified. Specifically, the glucan conversion at 72 hours of enzymatic hydrolysis of solid substrate was increased by 25.9% (from 41.6% to 65.7%) and 31.8% (from 51.7% to 83.5%) for SPORL-Po-A_1_B_4_ and SP-Lp-A_1_B_8_, respectively, by the complete application of the corresponding pretreatment hydrolysate. This can be ascribed to the role of LCCs in nonproductive binding reduction, which is similar to the effect of BSA treatment on enzymatic hydrolysis of cellulose in lignin containing substrates, as reported by Yang and Wyman [11]. Further, the role of LS in enzymatic hydrolysis of lignocellulose is quite similar to surfactant Tween 20, reported by Eriksson *et al*. [12]. Adding 5 g/L Tween 20 increased the glucan conversion of steam-pretreated spruce from 40% to 65%, while the adsorption of Cel was decreased from close to 90% to 65%. Compared to Tween 20 application [12], the increase of glucan conversion by LS addition was higher, particularly for substrate SP-Lp-A_1_B_8_.

**Table 2:** Chemical components of untreated wood chips, solid substrates, and pretreatment hydrolysate

**Untreated wood chips**		**Solid substrates**		**Pretreatment hydrolysate (mg/mL)**	
**Poplar (100 g)**	**Lodgepole pine (100 g)**	**SP-Po-A**_**1**_**B**_**4 **_**(73.5 g)**	**SP-Lp-A**_**1**_**B**_**8 **_**(58.6 g)**	**SP-Po-A**_**1**_**B**_**4 **_**(300 mL)**	**SP-Lp-A**_**1**_**B**_**8 **_**(300 mL)**
					
Klason lignin 23.5%	Klason lignin 28.6%	Klason lignin 26.2%	Klason lignin 34.7%	LS as Klason lignin 12.5	LS as Klason lignin 27.5
Glucan 40.7%	Glucan 41.9%	Glucan 48.4%	Glucan 57.4%	**Mono- and oligosaccharides**	**Mono- and oligosaccharides**
Xylan 16.4%	Xylan 5.5%	Xylan 4.38%	Xylan 1.5%	Glucose 3.97	Glucose 9.83
Mannan 3.8%	Mannan 11.7%	Mannan 1.48%	Mannan 0.6%	Xylose 41.00	Xylose 8.07
		Sulfonic acid groups	Sulfonic acid groups	Mannose 4.39	Mannose 21.88
				**Inhibitors**	**Inhibitors**
		4.22 mg/g substrate	10.85 mg/g substrate		
		Zeta potential at pH 4.8	Zeta potential at pH 4.8	Formic acid 24.58	Formic acid 17.05
				Acetic acid 10.65	Acetic acid 18.66
		−18.5 mv	−21.3 mv	Furfural 1.97	Furfural 4.17
				HMF 0.19	HMF 5.00

HMF, hydroxymethylfurfural; LS, lignosulfonate.

## Conclusions

This study revealed the formation of LCCs and their role in nonproductive binding mitigation during enzymatic hydrolysis of lignocellulose. LS, the byproduct of SPORL pretreatment, behaves as a polyelectrolyte to form LCCs with Cel. It was observed that the sulfonate acid groups on LS tended to associate to the oppositely charged groups on Cel, mainly the amino group. The results showed the complexation process was significantly affected by the ratio of LS to Cel. The elevated ratio of LS to Cel was constructive to the stability phase because of the increased surface potential and decreased particle size. Further, compared to Cel, LCCs were more negatively charged, and thereby had the ability of weakening the nonproductive binding of Cel to lignin due to the enlarged electrostatic repulsion. Isotherms of Cel adsorption showed the Cel binding by lignin was reduced from 182 mg/g to 88 mg/g by addition of LS. The LCCs-induced reduction of nonproductive binding was also reflected by the enhancement of glucan conversion with LS application. The experimental results showed that the glucan conversion of solid substrate of poplar and lodgepole pine was greatly increased by 25.9% and 31.8%, respectively, with the complete addition of the corresponding pretreatment hydrolysate.

## Materials and methods

### Materials

Novozymes Cellulosic Ethanol Enzyme Kit containing Cel (NS22086), β-glucosidase (NS22118), and hemicellulase (NS22002) were generously provided by Novozymes A/S (Bagsværd, Denmark). The molecular weight of Cel was assumed to be 50 kDa according to pertinent literature [45,46]. Wood logs of 6-year-old poplar were harvested from natural stands growing in the south region of Shandong province, China (N35°42′5.03″, E118°42′40.62″), and 100-year-old lodgepole pine from the Arapaho Roosevelt National Forest, CO, USA (N40°24′20.12″, W105°35′37.54″).

### SPORL pretreatment

SPORL pretreatment was carried out in a laboratory pulping digester, as described in our previous study [40]. Approximately 150 g of od wood chips were loaded. The procedures and conditions of SPORL pretreatment are listed in Table 3. After pretreatment, the wood chips were separated from the hydrolysate and then refined, as previously described [47]. The size-reduced solids were washed with deionized water to remove soluble degradation products for solid substrate production, that is, SP-Po-A_1_B_4_ and SP-Lp-A_1_B_8_. The Klason lignin content and polysaccharide content in the untreated wood and solid substrates (listed in Table 2) were determined according to the standard method of TAPPI T222 and literature [48], respectively. The majority of hemicellulose was removed by SPORL pretreatment as indicated by the changes of xylan and mannan content, which would facilitate the catalysis of cellulose. Most lignin remained in the solid substrate, but the sulfonation modification of lignin makes the solid substrate negatively charged, as suggested by the sulfonic acid groups and zeta potential in Table 2. The soluble degradation products in pretreatment hydrolysate listed in Table 2 were determined by HPLC according to the study of Luo *et al*. [49]. Clearly, the hydrolysate not only contains sugars and LS, but a lot of inhibitors in high concentration.

**Table 3:** List of pretreated lignocellulosic substrates studied with conditions for substrate production

**Wood species**	**Sample label**	**Chemical dosage on wood (weight %)**	**Wood to liquor ratio (w/v)**	**Temperature (°C)**	**Duration (minutes)**	**Disk gap for size reduction (mm)**	**Washing**
Poplar	SP-Po-A_1_B_4_	H_2_SO_4_: 1.1	1:3	175	25	0.25	Yes
		NaHSO_3_: 4					
Lodgepole pine	SP-Lp-A_1_B_8_	H_2_SO_4_: 1.1	1:3	185	25	0.25	Yes
		NaHSO_3_: 8					

### LS separation

The LS used in this study was isolated from the SPORL hydrolysate of poplar by membrane filtration according to the study of Madad *et al*. [50]. The poplar hydrolysate was filtered with 0.42 μm membrane. The obtained permeate was then subjected to diafiltration with 5 kDa MWCO membrane to remove impurities, such as salts and degradation products. Thereafter, the retentates inside the dialysis tube mainly contained LS and were used as pure LS. The molecule weight distribution of LS was measured by using size exclusion chromatography analysis (HPLC (LaChrom, Merck, Darmstadt, Germany) equipped with a Superdex 200 HR 10/30 column (GE Healthcare, Little Chalfont, UK)). The results showed a wide molecular weight distribution, with a number average molecule weight (M_n_) of 4,200 g/Mol and polydispersity of 1.53.

### LCCs characterization by DLS analysis

The solution of Cel was filtered by 0.22 μm syringe membranes (Millipore, Billerica, MA, USA) to remove microbial debris for sample preparation. Samples of Cel, LS, and LCCs were prepared at 50 mM sodium acetic buffer at a wide range of pH values, and 25°C for size and zeta potential measurements by DLS analyzer equipped with a laser Doppler microelectrophoresis (Zetasizer Nano ZS90, Malvern Instruments, Malvern, UK). In addition, LCCs were prepared at various molar ratios of Cel to LS to investigate the colloidal size and zeta potential, and thereafter to estimate the stability phase of LCCs, which can be reflected by the formation of aggregates and precipitates.

### Enzymatic hydrolysis

Enzymatic hydrolysis was conducted at 2% substrate solids (w/v) in pH 4.8 buffer solution on a shaker/incubator at 50°C and 200 rpm. Hydrolysate was sampled periodically for glucose concentration using HPLC (LC-20 T equipped with a column SCR-101C (Shimadzu, Kyoto, Japan)). Each data point was the average of two replicates. The average relative standard deviation was approximately 0.5%.

### Preparation of hydrolysis lignin residues

The hydrolysis lignin residues were prepared enzymatically from solid substrates of poplar according to Lou *et al*. [51]. The enzymatic hydrolysis of substrate was conducted twice using 4.0 g SP-Po-A_1_B_4_ at solid loading of 2% (w/v), 50°C, and pH 5.2 for 48 hours, with a relatively high Cel dosage of 20 FPU/g substrate and hemicellulase supplement of 0.7 mL/g substrate. After enzymatic hydrolysis, the centrifuging at 10,000 rpm and lyophilization at −42°C were used to gain lignin residues, which was then grinded in a quartz mortar to produce a uniform particle size to meet the requirement of Cel adsorption.

### Cel adsorption by hydrolysis lignin residues

Cel adsorption experiments were conducted at pH 5.2 and 25°C. The adsorption system consisted of 1.0 mL hydrolysis lignin residues solution (40 mg/mL), 0.5 mL Cel solution, and 0.5 mL buffer for control that was without LS addition or 0.5 mL LS solution (4.5 mg/mL). The initial concentrations of Cel were 0, 0.08, 0.4, 1.2, 1.6, and 2.4 mg/mL. After incubation for 1 hour, the free Cel in supernatant from centrifugal separation at 5,000 rpm for 5 minutes was measured according to Bradford protein assay [52]. The amount of the bound Cel on the hydrolysis lignin residues was calculated by subtracting the amount of the free Cel in the supernatant from the total amount of Cel applied initially. The Langmuir model equation (1) was used to fit the adsorption isotherm data. The maximum Cel adsorption capacity Q_s_ (mg protein/g lignin) and the Cel adsorption equilibrium constant *k* (mL/mg protein) were determined accordingly:

(1) $$\frac{1}{Q_{e}}=\frac{1}{Q_{s}}+\frac{1}{Q_{s}\cdot k}\cdot \frac{1}{C_{e}}$$

in which Q_e_ is the absorbed Cel at equilibrium (mg protein/g lignin) and C_e_ is the concentration of free Cel (mg protein/mL).

## Abbreviations

BSA: Bovine serum albumin; CBU: Cellobiase unit; Cel: Cellulase; DLS: Dynamic light scattering; FPL: Forest Products Laboratory; FPU: Filter paper unit; HMF: Hydroxymethylfurfural; HPLC: High-performance liquid chromatography; LCC: Lignosulfonate-cellulase complex; LS: Lignosulfonate; MW: Molecular weight; MWCO: Molecular weight cut off; od: Oven dry; PAMPS: Polyacrylamidomethylpropyl sulfonate; pI: Isoelectric point; SPORL: Sulfite pretreatment to overcome recalcitrance of lignocellulose; SSCombF: Simultaneous saccharification and combined fermentation; TAPPI: Technical Association of the Pulp and Paper Industry; USDA: United States Department of Agriculture.

## Competing interests

The authors declare that they have no competing interests.

## Authors’ contributions

ZW conducted most of the experiments and wrote the entire manuscript. Some initial research was directed by JZ at the United States Department of Agriculture (USDA) Forest Products Laboratory (FPL). ZS conducted the glucose measurements. YF conducted the DLS analysis with the assistance from JJ and FY. MQ provided some suggestions on experiment design and manuscript writing. All authors read and approved the final manuscript.
